# Supplementary material for: Influence of Material Parameter Variability on the Predicted Coronary Artery Biomechanical Environment via Uncertainty Quantification
Source: ArXiv. 2024 Jan 26:arXiv:2401.15047v1. Preprint. [Version 1] (PMC10854278)
Supplement: Supplement 1 [file NIHPP2401.15047v1-supplement-1.pdf]

Supplementary Material

Article title: Influence of Material Parameter Variability on The Predicted Coronary Artery Biomechanical Environment via Uncertainty Quantification

Journal name: Biomechanics and Modeling in Mechanobiology

Author names: Caleb C. Berggren, David Jiang, Y.F. Jack Wang, Jake A. Bergquist, Lindsay C. Rupp, Zexin Liu, Rob S. MacLeod, Akil Narayan, Lucas H. Timmins

Corresponding author: Lucas H. Timmins, Texas A&M University, lucas.timmins@tamu.edu

Supplementary Figure 1

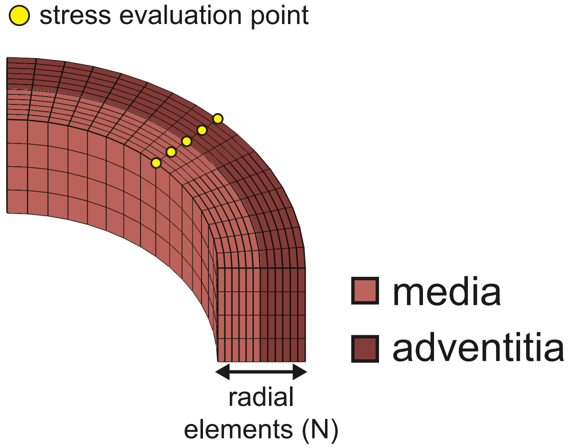

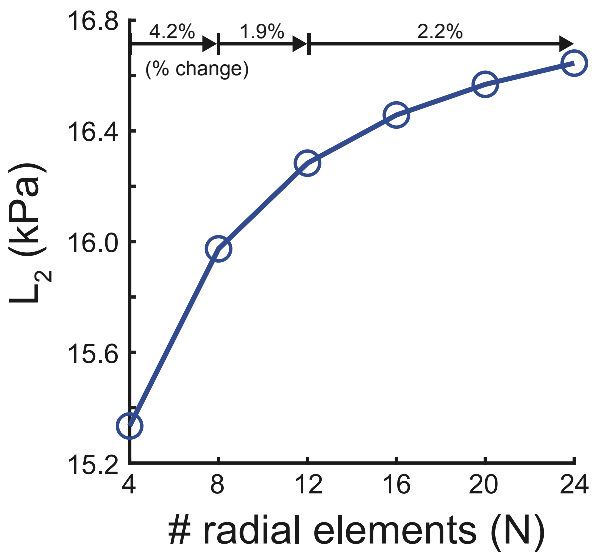

Mesh convergence study for the idealized artery model. The 2-norm criteria (L^2) for the 1st principal stress were evaluated at corresponding positions for radial mesh densities of 4, 8, 12, 16, 20, and 24 elements (equal number of elements in each layer). A change from 12 to 24 radial elements resulted in a change in L_2 for the 1st principal stress of <2.5%.

Supplementary Figure 2

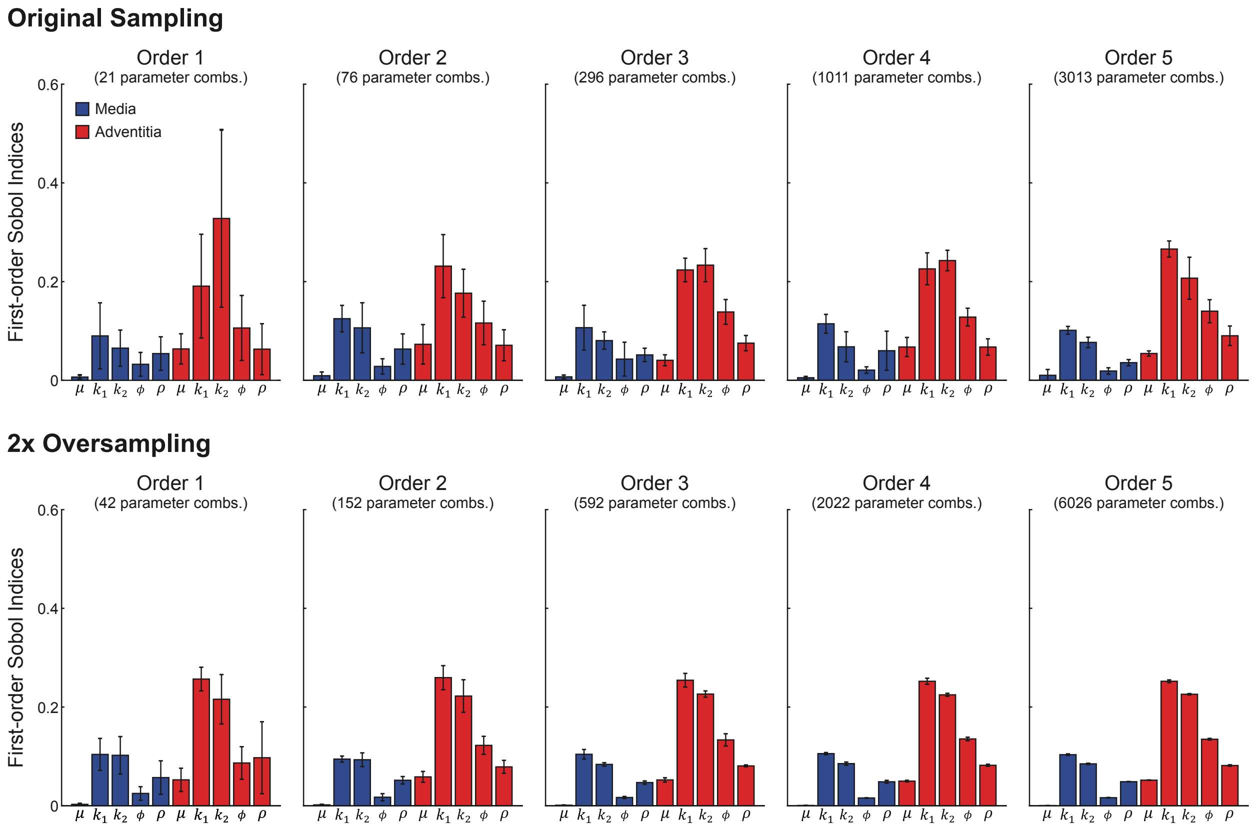

First-order Sobol indices in medial and adventitial layers at different parameter sampling rates. Original sampling (top row) and 2x oversampling (bottom row) across orders 1 through 5, with 5 runs for each order. Data are reported as mean ± standard deviation.

Supplementary Figure 3

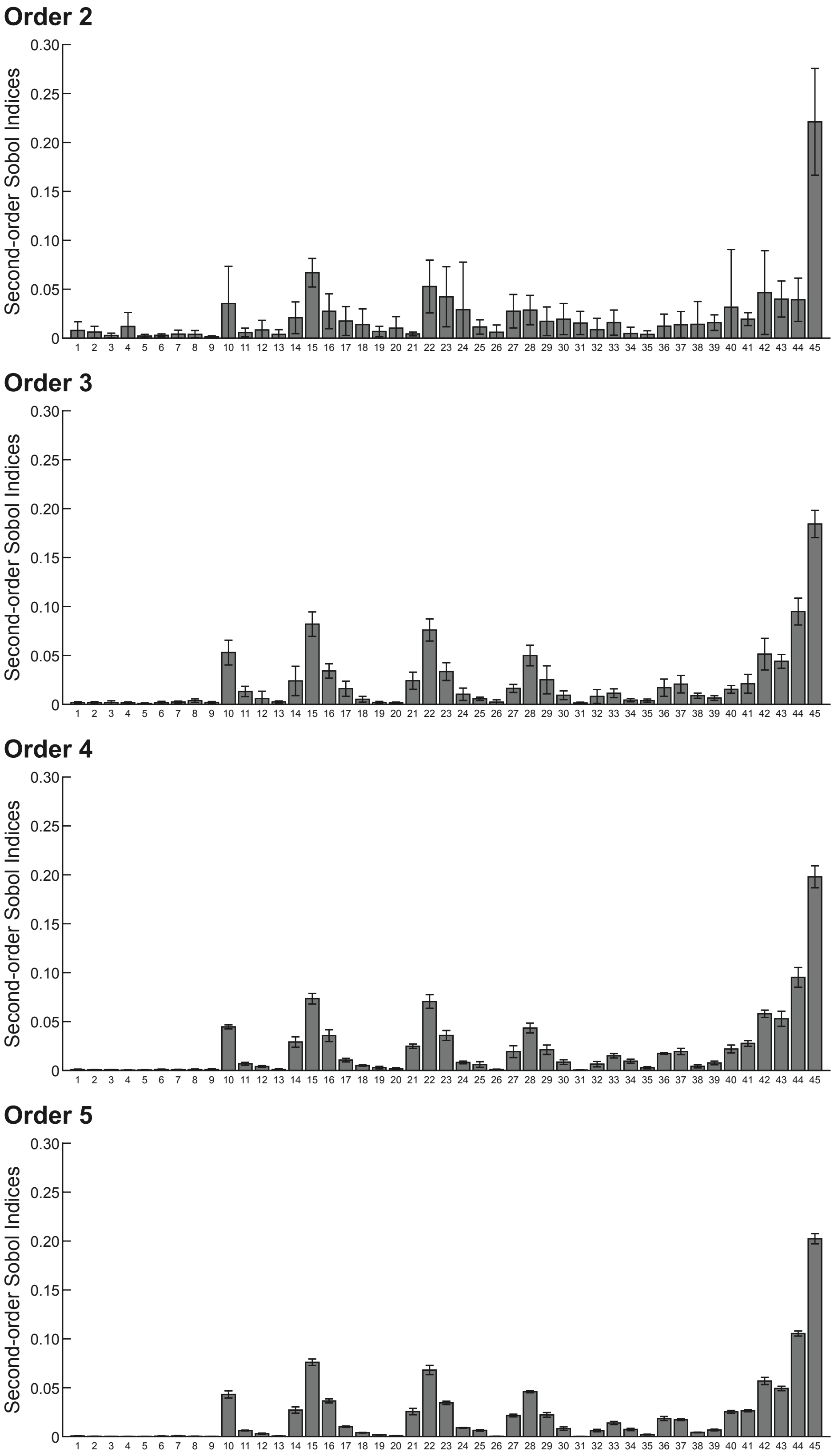

Second-order Sobol indices (i.e., binary interactions) across orders 2 through 5, with 5 runs for each order. Note that binary interactions cannot be evaluated at order 1. Data are reported as mean $\pm$ standard deviation.

X-axis legend (M: media, A: adventitia)

1: M,μ - M,k_1
2: M,μ - M,k_2
3: M,μ - M,ρ
4: M,μ - M,ϕ
5: M,μ - A,μ
6: M,μ - A,k_1
7: M,μ - A,k_2
8: M,μ - M,ρ
9: M,μ - A,ϕ
10: M,k_1 - M,k_2
11: M,k_1 - M,ρ
12: M,k_1 - M,ϕ
13: M,k_1 - A,μ
14: M,k_1 - A,k_1
15: M,k_1 - A,k_2
16: M,k_1 - A,ρ
17: M,k_1 - A,ϕ
18: M,k_2 - M,ρ
19: M,k_2 - M,ϕ
20: M,k_2 - A,μ
21: M,k_2 - A,k_1
22: M,k_2 - A,k_2
23: M,k_2 - A,ρ
24: M,k_2 - A,ϕ
25: M,ρ - M,ϕ
26: M,ρ - A,μ
27: M,ρ - A,k_1
28: M,ρ - A,k_2
29: M,ρ - A,ρ
30: M,ρ - A,ϕ
31: M,ϕ - A,μ
32: M,ϕ - A,k_1
33: M,ϕ - A,k_2
34: M,ϕ - A,ρ
35: M,ϕ - A,ϕ
36: A,μ - A,k_1
37: A,μ - A,k_2
38: A,μ - A,ρ
39: A,μ - A,ϕ
40: A,k_1 - A,k_2
41: A,k_1 - A,ρ
42: A,k_1 - A,ϕ
43: A,k_2 - A,ρ
44: A,k_2 - A,ϕ
45: A,ρ - A,ϕ
